# Internalizing and Externalizing Behaviors Share a Common Predictor: the Effects of Early Maladaptive Schemas Are Mediated by Coping Responses and Schema Modes

**DOI:** 10.1007/s10802-017-0386-2

**Published:** 2018-01-12

**Authors:** Marjolein F. van Wijk-Herbrink, David P. Bernstein, Nick J. Broers, Jeffrey Roelofs, Marleen M. Rijkeboer, Arnoud Arntz

**Affiliations:** 1 Conrisq Group, Post Box 1, 6670 AA Zetten, The Netherlands; 2 Department of Clinical Psychological Science, Maastricht University, Post Box 616, 6200 MD Maastricht, The Netherlands; 3 Department of Methodology and Statistics, Maastricht University, Post Box 616, 6200 MD Maastricht, The Netherlands; 4 Department of Clinical Psychology, University of Utrecht, Post Box 80140, 3508 TC Utrecht, The Netherlands; 5 Department of Clinical Psychology, University of Amsterdam, Post Box 15933, 1001 NK Amsterdam, The Netherlands

**Keywords:** Early maladaptive schemas, Coping, Schema modes, Adolescents, Internalizing behavior problems, Externalizing behavior problems

## Abstract

We investigated the relationships of adolescents’ internalizing and externalizing behaviors with their early maladaptive schemas (EMS), coping responses, and schema modes. We focused on EMS related to experiences of disconnection and rejection that comprise vulnerable emotions, such as shame, mistrust, deprivation, abandonment, and isolation/alienation. This cross-sectional study included a total of 699 adolescents (combined clinical and non-referred sample) who were 11 to 18 years old (*M* = 14.6; *SD* = 1.6), and of which 45% was male. All participants completed self-report questionnaires on EMS, coping responses, schema modes, and behavior problems. We aimed to clarify the relationships between these variables by testing mediation, moderation, and moderated mediation models. In general, coping responses functioned as mediators rather than moderators in the relationships between EMS and schema modes. Furthermore, EMS regarding experiences of disconnection and rejection were related to both internalizing and externalizing behavior problems, and coping responses and schema modes mediated these effects. In conclusion, although adolescent internalizing and externalizing behavior problems manifest quite differently, they seem related to the same EMS.

There is a growing body of literature demonstrating relationships between Young (1994) early maladaptive schemas (EMS) and later emotional and behavioral problems in adolescents (e.g., Calvete and Orue 2012; Muris 2006; Van Vlierberghe and Braet 2007; Van Vlierberghe et al. 2010). EMS are repeating, self-defeating patterns, consisting of cognitions, affects, memories, and physiological reactions (Young 1994). They develop in early childhood through the interaction of adverse childhood experiences and the child’s innate temperament. EMS bias processing of social information, evoking negative emotions and dysfunctional thoughts, which may ultimately result in internalizing and externalizing behavior problems.

Nevertheless, a number of critical questions about these relationships remain unanswered. First, are there relationships between specific EMS and specific kinds of behavior problems, such as internalizing versus externalizing behaviors? Or, is it possible that the same EMS can result in different behavior problems, depending on other factors, such as coping responses (i.e., moderating models)? Further, what are the theoretically indicated intervening variables between EMS and internalizing versus externalizing behavior problems (i.e., mediating models)?

## EMS and Internalizing versus Externalizing Behavior Problems

Several cross-sectional studies have tried to clarify the relationships between EMS and internalizing versus externalizing behavior problems in adolescence. Van Vlierberghe and Braet (2007) found that 45% of the variance in internalizing problems was explained by the EMS Social isolation (the expectation that one will never fit in) and Vulnerability to harm/illness (the expectation that a catastrophe can happen any time and that there is nothing one can do about it). They also found that 19% of the variance in externalizing problems was explained by the EMS Entitlement/grandiosity (the perception that one is superior to others and entitled to special rights) and Dependence/incompetence (the belief that one is unable to handle everyday responsibilities without help). Other studies (Muris 2006; Van Vlierberghe et al. 2010) found sets of schemas that uniquely contributed to certain types of problems, such as depressive symptoms, anxiety symptoms, and disruptive behaviors (explained variance ranging from 0.38 to 0.52). However, these sets of schemas did not converge across the studies.

Thus, the existing literature is inconsistent regarding the nature of these relationships, which may in part be due to differences in study populations, measures, and other methodological differences. Nevertheless, all studies reported some evidence that EMS related to experiences of disconnection and rejection are predictive of both internalizing and externalizing problems. Such EMS include Abandonment (i.e., expecting to be abandoned in close relationships), Mistrust/Abuse (i.e., expecting to be mistreated by others), Emotional deprivation (i.e., expecting that others will not meet one’s needs), Social isolation (i.e., feeling different from others; expecting to never fit in), and Defectiveness/Shame (i.e., perceiving the self as inferior, unwanted, or unlovable). EMS concerning experiences of disconnection and rejection are related to attachment difficulties arising in the early years, and evoke emotions of shame, mistrust, deprivation, abandonment, and isolation/alienation. It may be that more complex models, for example moderating or mediating models, are necessary to clarify the relationships between EMS regarding experiences of disconnection and rejection, and internalizing versus externalizing behaviors.

## Schema Theory: an Explanatory Model of Behavior

Schema theory (Young et al. 2003) provides a model for the relationship between EMS and behavior. Young and colleagues theorized that EMS, when triggered in different situations, evoke intense emotions (e.g., shame, sadness, fear, or anger) as well as attempts at coping. Coping responses (surrender, avoidance, and overcompensation) to activated EMS are theorized to result in schema modes, which are transient emotional-cognitive-behavioral states. Whereas EMS are trait-like entities, schema modes are the state variants of EMS. For example, a Defectiveness schema (i.e., perceiving the self as inferior, unwanted, or unlovable), combined with a surrendering coping response, could produce an emotional-cognitive-behavioral state involving giving in to painful feelings of inferiority and sadness, known as Vulnerable Child mode. In contrast, the same Defectiveness schema, coupled with an Overcompensating coping response, could produce a state of arrogance and superiority, known as Self-Aggrandizer mode (see Table 1).

**Table 1:**
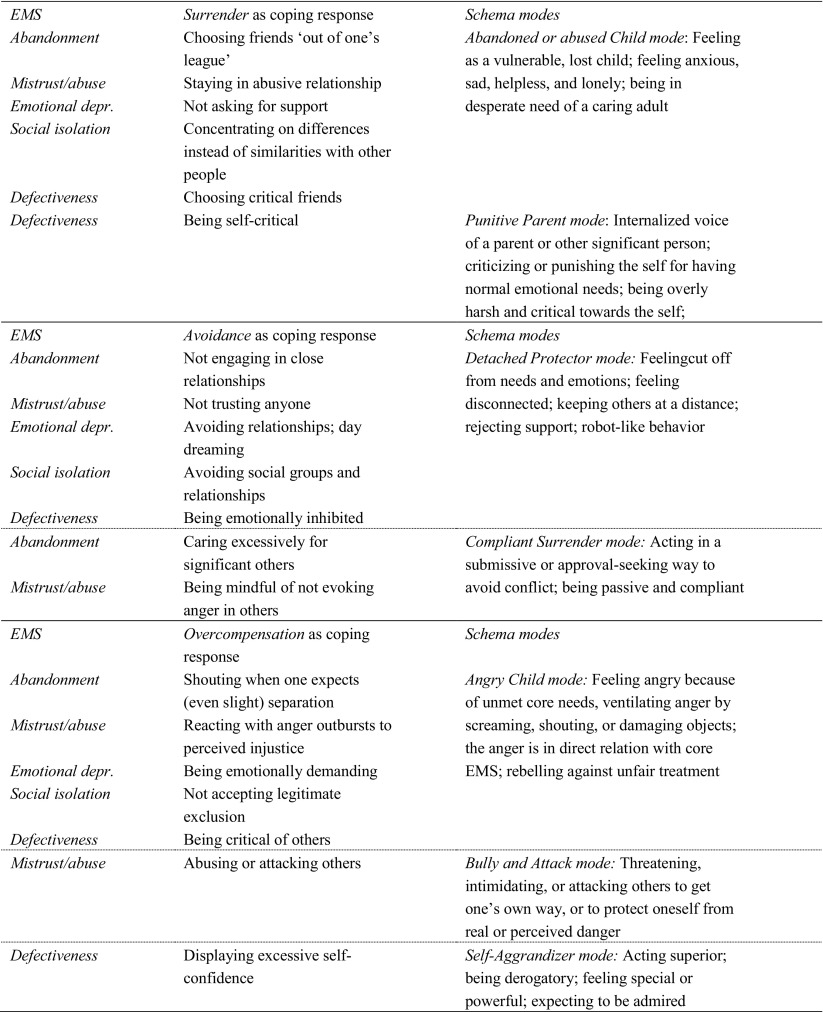
Theorized relationships between EMS, schema coping, and schema modes

Rijkeboer and Lobbestael (2012) tested the schema theory with a cross-sectional design in a large sample of adult patients (*N* = 1602). They found clear evidence for the mediating role of coping responses in the relationship between specific EMS and schema modes for almost every combination that they tested. They split their sample in half to cross-validate their findings. In both samples, they found significant indirect effects of specific EMS on specific schema modes through coping (explained variance ranged from 0.34 to 0.74). Their findings suggest that EMS can result in different types of emotional states, when mediated by different coping responses. Nevertheless, they did not examine these relationships in adolescence, when behavior problems often first appear, nor did they investigate the relationships between the schema theory constructs and internalizing versus externalizing behavior problems. A recent study in adolescents showed that surrendering coping, internalizing modes (e.g., Vulnerable Child mode), and internalizing behavior problems were all related to each other, and that overcompensatory coping, externalizing modes (e.g., Angry Child mode), and externalizing behavior problems were also related to each other (Van Wijk-Herbrink et al. 2017b). This study also showed that avoidant coping was not, or only weakly, related to such schema modes and behaviors.

## Present Study

In the present study, we aimed to test relationships between the schema theory constructs and behavior problems in adolescents. Adolescence is a period when internalizing and externalizing behavior problems often become manifest, and where early intervention may prevent the development of more severe or chronic problems. Studying these issues in adolescents would not only contribute to the development of more adequate theoretical models of these phenomena, but might also point the way to more effective interventions.

We used a combined sample of clinical and non-referred adolescents in order to benefit from the large sample size and to increase variance. Combining the samples is in line with theory and research, suggesting that the schema theory constructs are consistent dimensions occurring in both clinical and healthy samples (e.g., Rijkeboer and Lobbestael 2016; Rijkeboer and van den Bergh 2006; Roelofs et al. 2015; Van Vlierberghe et al. 2010; Van Wijk-Herbrink et al. 2017b). Consistent with the idea of dimensionality, these studies show differences in severity of these constructs between clinical and non-clinical samples. Nonetheless, the relationships between these constructs are thought to be the same for both groups: When EMS are triggered, certain coping responses may be adopted, resulting in certain schema modes.

We tested three possible models for the relationships between EMS, coping responses, and schema modes, and consequently tested models for the relationships between schema theory constructs and behavior problems. We used the schema theory combinations found by Rijkeboer and Lobbestael (2012), thereby focusing on the five EMS regarding experiences of disconnection and rejection. All combinations are illustrated in Table 1.

### Mediation

In an attempt to replicate the findings of Rijkeboer and Lobbestael (2012), we first tested whether coping is the mechanism through which EMS exert their effect on schema modes. From schema theory, we would expect EMS to have an effect on schema coping, and schema coping to have an effect on schema modes. We hypothesized that, for all combinations in Table 1, coping would mediate the relationship between EMS and schema modes (e.g., relationship between EMS Abandonment and Vulnerable Child mode goes through surrendering coping).

### Moderation

From schema theory, it could also be that the effect of EMS on schema modes is dependent on schema coping: When EMS are triggered, the activation of schema modes may rely on the level of specific coping styles that are adopted. Thus, we hypothesized that, for all combinations in Table 1, coping would moderate the relationship between EMS and schema modes (e.g., relationship between EMS Abandonment and Vulnerable Child mode exists only (or is stronger) if surrendering coping is high).

### Moderated Mediation

As a third step, we tested whether schema coping is both a mediating and moderating variable at the same time. It may be that EMS activate schema modes through coping, but only when a certain level of dysfunctional coping is achieved. Thus, for all combinations in Table 1, we hypothesized that coping both mediates and moderates the relationships between EMS and schema modes (e.g., relationship between EMS Abandonment and Vulnerable Child mode goes through surrendering coping, but only (or more strongly) if surrendering coping is high).

### Models for Schema Theory Constructs and Internalizing versus Externalizing Problems

**Fig. 1:**
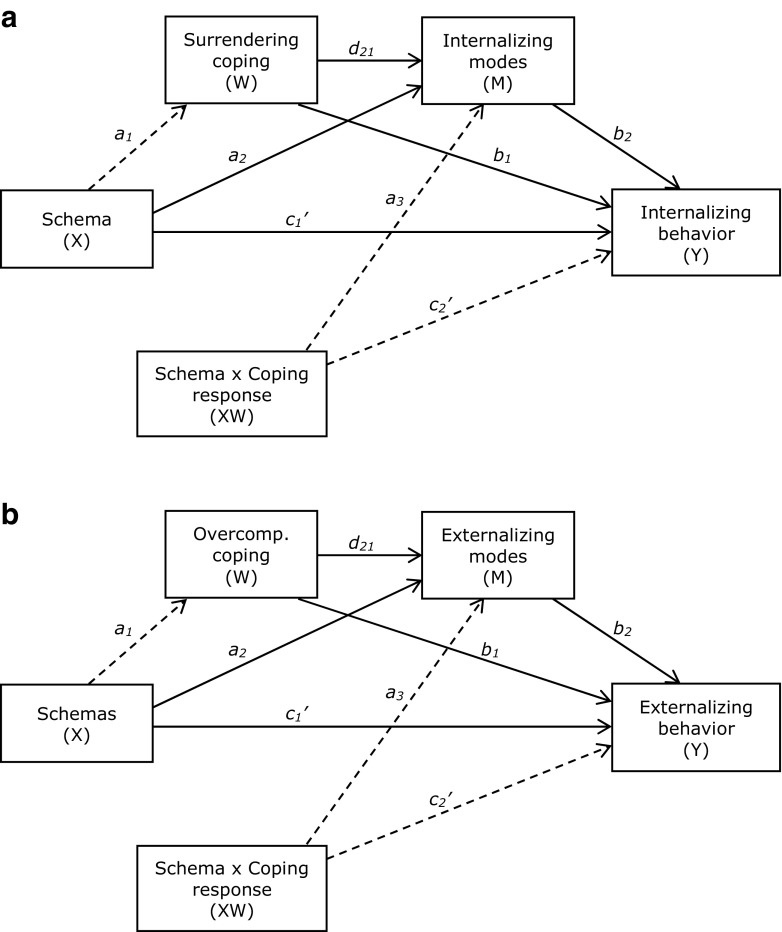
Hypothesized models the (conditional) indirect and direct effects of Disconnection and Rejection EMS on internalizing behavior problems (1**a**) versus externalizing behavior problems (1**b**). Depending on the results of Step 1 to 3, paths *a*_*1,*_ or *a*_*3*_ and *c’*_*2*_ (striped arrows) may be removed. If path *a*_*1*_ is removed, this will result in a moderated mediation model hypothesizing surrendering and overcompensatory coping as moderators of the indirect (through internalizing vs. externalizing modes) and direct effects of EMS on internalizing versus externalizing behavior problems. If paths a3 and c2’ are removed, this will result in a multiple serial mediation model in which surrendering versus overcompensatory coping is treated as a first mediator, and internalizing versus externalizing modes as a second mediator of the effect of EMS on internalizing versus externalizing behavior problems

As a final step of the present study, we examined the nature of the relationships between EMS, schema coping, schema modes, and internalizing versus externalizing behavior problems. We hypothesized that EMS would predict behavior problems through schema coping (and/or dependent on schema coping) and through schema modes (see Fig. 1). We included only the schema coping styles surrender and overcompensation, not avoidance, because previous research showed that avoidance was not or only weakly correlated with internalizing or externalizing behavior problems after correcting for other coping responses (Van Wijk-Herbrink et al. 2017b).

## Method

### Participants and Procedure

This study was based on the same dataset (*N* = 699) as our previous study on the validation of schema coping and schema modes in adolescents (Van Wijk-Herbrink et al. 2017b), to which we refer for more details about the sample, procedure, and instruments used. Participants from the non-referred sample were recruited from a secondary school in the Netherlands. All 1600 pupils of this school were approached for participation, and 36% of them (and their parents) gave informed consent, resulting in a non-referred sample of 577 adolescents. This sample included 242 males and 335 females between 11 and 18 years old (*M* = 14.4, *SD* = 1.7), of which 98% was of Dutch origin.

The clinical sample was recruited from two residential treatment centers with open and secure treatment groups for adolescent patients with severe behavior problems. The questionnaires used in this study were administered as a standard clinical procedure, and were retrieved from the dossiers of all patients who were in treatment at the time of recruiting the non-referred sample. Sixty-nine percent of the dossiers contained completed questionnaires. Consent for the anonymous use of these data for research purposes was included in the written consent for clinical treatment, which was given by both patients and their parents. The Ethical Committee of Maastricht University in the Netherlands approved this procedure, as well as all other procedures of this study. The clinical sample included 70 males and 52 females between 12 and 18 years old (*M* = 15.5, *SD* = 1.2), of which 84% was of Dutch origin. Although these patients are usually admitted for their externalizing behavior problems, they also show high rates of internalizing behavior problems (Nijhof et al. 2011). Most prevalent DSM-IV (American Psychiatric Association, 2000) chart diagnoses of the patients included Disruptive Behavior Disorders (67%), emerging Personality Disorders or Personality Disorder traits (58%), Substance Abuse Disorder (31%), Attention Deficit and Hyperactivity Disorder (26%), Autism Spectrum Disorder (19%), Post-Traumatic Stress Disorder (17%), Reactive Attachment Disorder (17%), and Mood Disorders (14%).

### Instruments

#### EMS

The Young Schema Questionnaire for Adolescents (YSQ-A; Van Vlierberghe et al. 2004) reflects 15 EMS as defined by Young (1994). Each EMS is represented by five items to be rated on a 6-point Likert scale (1 = *not at all true* through 6 = *totally true*). In our study, we administered only the items from the EMS Abandonment (e.g., “I am concerned that the people I care about will abandon me”), Mistrust/Abuse (e.g., “I think that people will take advantage of me”), Emotional deprivation (e.g., “I have never received love and attention”), Social isolation (e.g., “I don’t fit in”), and Defectiveness/Shame (e.g., “No boy or girl I like could love me once he or she gets to know my flaws”), which have consistently been shown to load on a higher-order factor called the Disconnection and Rejection domain in both adults (Lee et al. 1999; Schmidt et al. 1995;) and adolescents (Muris 2006; Van Vlierberghe et al. 2010). Studies have shown that the internal consistency of the Disconnection and Rejection scales is good in adults (0.91 to 0.96, *M* = 0.93; Rijkeboer and van den Bergh 2006; Schmidt et al. 1995; Welburn et al. 2002) and acceptable in adolescents (0.70 to 0.86, *M* = 0.77; Muris 2006; Van Vlierberghe et al. 2010). In the current sample, internal consistency ranged from 0.83 to 0.90 (*M* = 0.86) for the EMS scales of the Disconnection and Rejection domain, and the total internal consistency for the domain was 0.94. Test-retest reliabilities are available only for adults and range from 0.67 to 0.82 (*M* = 0.75) over a 3-week period (Schmidt et al. 1995).

In both adults and adolescents, YSQ scales have been shown to discriminate between clinical and non-clinical populations (Rijkeboer and van den Bergh 2006; Rijkeboer et al. 2005; Van Vlierberghe et al. 2010). Furthermore, regression analyses supported the construct validity of the YSQ by revealing that EMS account for 50 to 63% of the variance in depression symptoms and for 34 to 50% of the variance in anxiety symptoms (Glaser et al. 2002; Schmidt et al. 1995; Van Vlierberghe et al. 2010), and, specifically in adolescents, for 44% of the variance in disruptive behavior (Van Vlierberghe et al. 2010).

#### Schema Modes

We used an 80-item version of the Schema Mode Inventory (SMI; Lobbestael et al. 2010) to measure schema modes. For this 80-item version of the Schema Mode Inventory, the five items with highest loadings on each schema mode were selected from the SMI, which originally constitutes 118 items (see Keulen-de Vos et al. 2015) to be rated on a 6-point Likert scale (1 = *never or hardly ever* through 6 = *always*). In the present study, we used mean scores on schema mode scales that have been shown to load on higher-order factors of Internalizing modes (Abandoned Child, Lonely Child, Punitive Parent, Compliant Surrenderer, and Detached Protector) and Externalizing modes (Angry Child, Enraged Child, Impulsive Child, Undisciplined Child, and Bully and Attack mode) in both adults (Keulen-de Vos et al. 2015) and adolescents (Roelofs et al. 2015; Van Wijk-Herbrink et al. 2017b). Additionally, we used mean scores on the Self-Aggrandizer mode (which loaded on the externalizing factor in the Keulen-De Vos study, but on a separate factor called Overachieving modes in the Van Wijk-Herbrink study) and mean scores on the Internalizing and Externalizing factors. The internalizing and externalizing factors have shown good internal consistency, with alpha values of 0.88 for both factors in adults (Keulen-de Vos et al. 2015) and values of 0.95 (internalizing) and 0.92 (externalizing) in the current sample (Van Wijk-Herbrink et al. 2017b). Internal consistencies for the individual schema modes used in the present study are comparable in adolescents and adults, ranging from 0.70 to 0.96, *M* = 0.86 (Lobbestael et al. 2010; Reiss et al. 2011; Roelofs et al. 2015; Van Wijk-Herbrink et al. 2017b). Four-week test-retest reliabilities ranged from 0.65 to 0.92, *M* = 0.83, in adults (Lobbestael et al. 2010).

Compared to healthy controls, both adult and adolescent patients have been shown to score higher on dysfunctional schema modes as used in the present study (Lobbestael et al. 2010; Reiss et al. 2011; Van Wijk-Herbrink et al. 2017b). Furthermore, studies showed that schema modes explain 56% of the variance in psychopathology and 35% of the variance in quality of life (Roelofs et al. 2015), and that they explain additional variance in Axis II disorders above Axis I disorders (*R*^*2*^ *change* ranging from 2.4 to 12.2, *M* = 9.2%; Lobbestael et al. 2010). Another study supporting the construct validity of schema modes (Van Wijk-Herbrink et al. 2017b) showed that Internalizing modes were positively associated with internalizing behaviors (*r* = 0.56) and negatively associated with externalizing behaviors (*r* = −0.19), whereas externalizing modes were positively associated with externalizing behaviors (*r* = 0.65) and negatively associated with internalizing behaviors (*r* = −0.19).

#### Schema Coping

The Schema Coping Inventory (SCI; Rijkeboer et al. 2010) consists of 12 items to be rated on a 7-point Likert-scale (1 = *totally disagree* through 7 = *totally agree*). In the present study, we used mean scores on the three coping scales: Surrender (e.g., “In case of difficulty, I tend to give up”), Avoidance (e.g., “It is best to switch off your feelings as much as possible”), and Overcompensation (e.g., “I tend to overrule and control others”). Rijkeboer and Lobbestael (2016) randomly split their total sample of 1602 adult patients in two, creating an exploration sample (*n =* 801) in which a model-generating procedure was followed (Jöreskog and Sörbom 1996), and a validation sample (*n =* 801) in which the found factor structure was cross-validated, using a strict confirmatory procedure. Using structural equation modeling, they found that all fit indices of the established three-factor model showed a good fit to the data in both samples (CFI > 0.97, NNFI > 0.96, SRMR < 0.044, and GFI > 0.95), and internal consistency values ranged from 0.75 to 0.86, *M =* 0.80. The three-factor structure was replicated in the adolescent clinical and non-referred samples that constitute the current sample of the present study, and high levels of measurement invariance between the subsamples were established (Van Wijk-Herbrink et al. 2017b). For the current sample, internal consistency values were considerably higher for the clinical adolescent sample (0.71–0.78, *M =* 0.75) than for the non-referred adolescent sample (0.61–0.67, *M =* 0.64; Van Wijk-Herbrink et al. 2017b).

In adults, regression analyses revealed that the coping scales were uniquely related to personality disorder traits. Positive associations (*p’*s *<* 0.001) were found for Surrender with dependent and depressive traits (γ’s > 0.28), for Avoidance with avoidant personality traits (γ = 0.49), and for Overcompensation with paranoid, narcissistic, passive-aggressive, and obsessive-compulsive traits (γ’s > 0.25; Rijkeboer and Lobbestael 2016). In adolescents, strong positive associations (*p’*s *<* 0.001) were found for Surrender with internalizing schema modes (*r* = 0.37 for Surrender) and internalizing behavior problems (*r* = 0.51), and for Overcompensation with externalizing modes (*r* = 0.19) and externalizing behaviors (*r* = 0.24). Somewhat weaker, but significant associations (*p’*s *<* 0.001) were found for Avoidance with internalizing schema modes (*r* = 0.16) and internalizing behavior problems (*r* = 0.10).

#### Behavior Problems

Participants filled out the Youth Self-Report (YSR; Achenbach and Rescorla 2001), rating items from the Internalizing and Externalizing problems scales as 0 (*not true*), 1 (*somewhat or sometimes true*), or 2 (*very true or often true*). Each scale was represented by 32 items, and scale scores were the sum of these items. The YSR has shown good psychometric properties in many different languages. The Internalizing and Externalizing scales of the Dutch version have high internal consistency (0.91–0.95) and test-retest reliability, and stability coefficients are 0.59 (Internalizing) and 0.60 (Externalizing) for a 2-year interval and 0.45 (Internalizing) and 0.46 (Externalizing) for a 4-year interval (Verhulst and Van der Ende 2013). In the current sample, the internal consistency was 0.93 for the Internalizing problems scale and 0.92 for the Externalizing problems scale. The Internalizng and Externalizing scales distinguish well between referred and non-referred youth (Verhulst and Van der Ende 2013). Achenbach and Rescorla (2001) showed that the Internalizing problems scale correlated with depressive disorders (*r* = 0.45–0.59), and that the Externalizing problems scale correlated with conduct disorder (*r* = 0.30–0.62).

### Statistical Analyses

Most participants completed the questionnaires through a secure web page that does not allow missing values. Only those few participants, who did not have access to internet (usually patients in high secure treatment units), filled out pen-and-paper questionnaires. Therefore, missing data occurred only occasionally, and were replaced by the mean of the other items belonging to the same scale (so that the missing data would not influence the scale scores).

We followed four steps to clarify the relationships between the constructs of schema theory and adolescent’s internalizing versus externalizing behavior problems. In Step 1, we conducted mediation analyses to replicate the findings of Rijkeboer and Lobbestael (2012) in our adolescent sample. In Step 2, we investigated the same relationships between EMS and schema modes, but this time we used moderation models rather than mediation models for the role of coping. If there was evidence for both models, we proceeded with Step 3 and tested an integrated model of moderated mediation (Hayes 2013), implicating that schema coping can act as a mediator and a moderator at the same time. In Step 4, we used only higher-order variables of EMS and schema modes, and added internalizing versus externalizing behavior problems to the models (See Fig. 1). Based on the results of Step 1–3, we decided whether to treat surrendering coping as a mediator, as a moderator, or both.

We hypothesized that the relationships between EMS, schema coping, and schema modes would be the same for adolescents from the clinical and non-referred samples. We tested this by adding group as a moderator to the analyses described in Step 1 and 2. In Step 1, we tested statistical significance of indexes of moderated mediation (which, for dichotomous moderators, test group differences in indirect effects; Hayes 2013). In Step 2, we tested statistical significance of 3-way interaction effects (which test group differences in the interaction between EMS and coping). If these indexes of moderated mediation and interaction effects were not statistically significant, we conducted the analyses of Step 1 to 4 on the combined sample of clinical and non-referred adolescents to benefit from the large sample size.

We tested all models in the four steps using the PROCESS macro (Hayes 2013) for SPSS (version 22), which is based on OLS regression analysis. The moderation analyses (not mediation analyses because of the bootstrapping technique) assume normal distribution of estimation errors of the dependent variables. Because the variables in our study (as in many other psychological studies) are not normally distributed, the estimation errors probably are also not normally distributed. Fortunately, violations of this assumption have been shown to have little effect on linear regression analysis (e.g. Edgell and Noon 1984; Havlicek and Peterson 1977). Other assumptions for OLS regression analyses, such as linearity, homoscedasticity, and independent errors were generally met. For all analyses, we reported model coefficients and direct, indirect, and interaction effects in unstandardized form in order to facilitate comparison with future studies using the same instruments. As a measure of the effect size of the mediated effect, we reported the kappa-squared index (*κ*^*2*^; Preacher and Kelley 2011) of the indirect effects. This *κ*^*2*^ is not interpreted relative to zero, but relative to how large the indirect effect could possibly be given the variances and correlations between the variables observed. For the interaction effects, we reported changes in the proportion of explained variance (*∆R*^*2*^) as a measure of the effect size of the moderated effect. For the complex moderated mediation models, no effect sizes are available yet (Hayes 2013). For multiple mediation models, we used the completely standardized indirect effect (CSE) as a measure of the effect size, because the kappa-squared (*κ*^2^) is not available for these models.

To test for statistical significance of indirect effects, we used bias-corrected bootstrap confidence intervals (based on 50,000 bootstrap samples) as calculated by PROCESS (Hayes 2013). If multiple mediators were used in the fourth step, we tested the significance of the differences between the indirect effects. Although the mediating variables were not measured on the same scale, the indirect effects through these variables can be meaningfully compared. After all, an indirect effect is defined as the amount by which two cases differing by one unit on X are estimated to differ on Y through the mediating variable, independent of other mediating variables (Hayes 2013). Therefore, the scaling of the mediating variables plays no role in the interpretation of the indirect effects.

Because in total, we planned to conduct 42 to 62 analyses,^1^ we applied a correction to the significance level based on the experimentwise error (Maxwell 1992). To achieve a conventional Type I error of 5% for each analysis, the experiment-wise error rate should be approximately 0.001 (i.e., 0.0009 for 62 analyses and 0.0012 for 42 analyses). Although this is a stringent significance level, the sample size is large enough to use this significance level in order to minimize chance findings, and still have enough power to detect small effects. A power analysis using G*Power (version 3.1.9.2; Faul et al. 2009) showed that with a sample size of 699 and a significance level of 0.001, we have 80% power to find an interaction effect with an effect size (f^2^) of 0.02 or larger in the moderation analyses. For the mediation analyses, we used the bias-corrected bootstrap method to detect indirect effects. Because conducting a power analysis for this method is rather complex, we used the MedPower program (Kenny 2017) which determines the power of the test of joint significance of paths a and b (MacKinnon et al. 2002). As the bootstrapping method has been demonstrated to have more power than the joint significance test to detect indirect effects (e.g., Fritz and MacKinnon 2007), the result of the MedPower analysis can be seen as a lower limit of the power of our mediation analyses. The MedPower analysis showed that a sample size of 499 is sufficient to achieve 80% power of detecting a significant effect at the 0.001 level, even if the regression coefficients of paths a and b are as low as 0.20. Our sample size of 699 is therefore unlikely to give power issues in the mediation analyses.

## Results

Results of Step 1 to 3 involving the relationships between specific EMS, coping, and schema modes are displayed in Table 2. Results of these steps involving the models with higher-order factors of EMS (Disconnection and Rejection EMS) and schema modes (Internalizing and Externalizing modes) are displayed in Table 3. All results are based on the combined sample of clinical and non-referred adolescents, because adding group as a moderator to the mediation and moderation analyses revealed no group differences.^2^ Thus, as hypothesized, the relationships between schemas, coping, and schema modes were consistent across the clinical and non-referred samples.

**Table 2:**
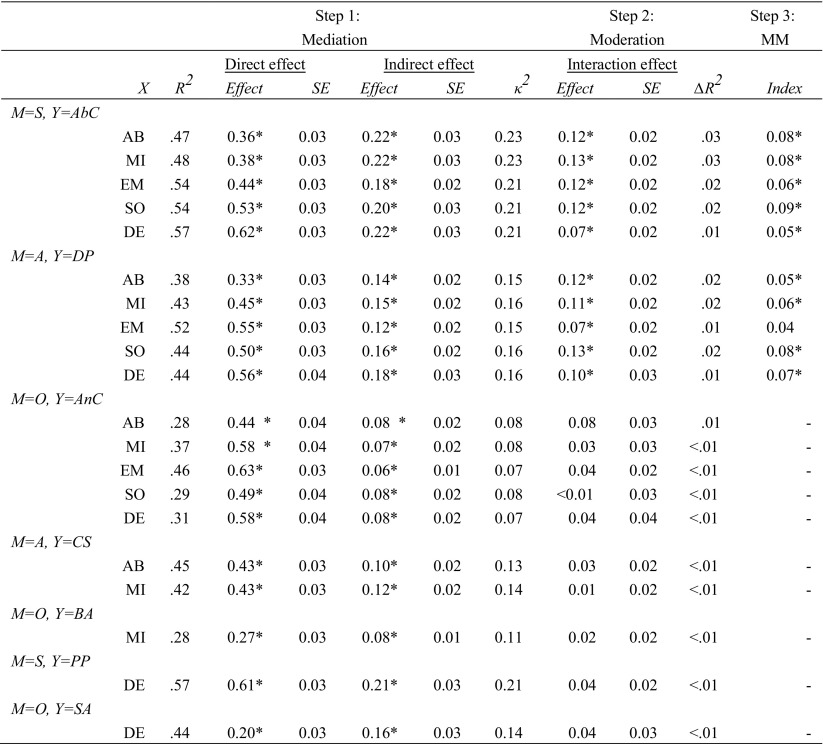
Effects of mediation, moderation, and moderated mediation analyses investigating the role of schema coping responses in the relationships between specific EMS and schema modes

X = independent variable (EMS); *R*^*2*^ = proportion of explained variance in the model with schema and coping response as predictors; *SE* = standard error; *∆R*^*2*^ = increase in *R*^*2*^ due to the interaction; *κ*^*2*^ = effect size of the indirect effect; MM = moderated mediation; *M* = mediating/moderating variable (schema coping); *Y* = dependent variable (schema mode). AB = Abandonment; MI = Mistrust/Abuse; EM = Emotional deprivation; SO = Social isolation; DE = Defectiveness; S = Surrender, A = Avoidance, O = Overcompensation. AbC = Abandoned child, DP = Detached protector, AnC = Angry child, CS = Compliant Surrenderer, BA = Bully and attack, PP = Punitive parent, SA = Self-aggrandizer

*significant at the 0.001 level: 99.9% confidence intervals (direct effects and interaction effects) or 99.9% bias-corrected bootstrap confidence intervals (indirect effects and index of moderated mediation) did not straddle zero

**Table 3:**
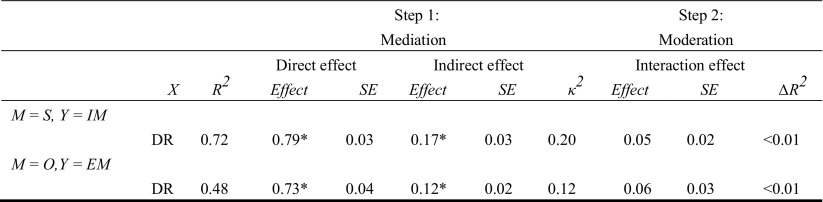
Effects of mediation and moderation analyses investigating the role of schema coping responses in the relationship between disconnection and rejection EMS and internalizing versus externalizing modes

*R*^*2*^ = proportion of explained variance in the model with schema and coping response as predictors; *∆R*^*2*^ = increase in *R*^*2*^ due to the interaction. *κ*^*2*^ = effect size of the indirect effect. DR = EMS from the domain of Disconnection and Rejection. S = Surrender; O = Overcompensation. IM = Internalizing modes, EM = Externalizing modes

*significant at the 0.001 level: 99.9% confidence intervals (direct effects and interaction effects) or 99.9% bias-corrected bootstrap confidence intervals (indirect effects) did not straddle zero

### Role of Coping in the Relationship between EMS and Schema Modes

#### Step 1: Simple Mediation Analyses

**Fig. 2:**
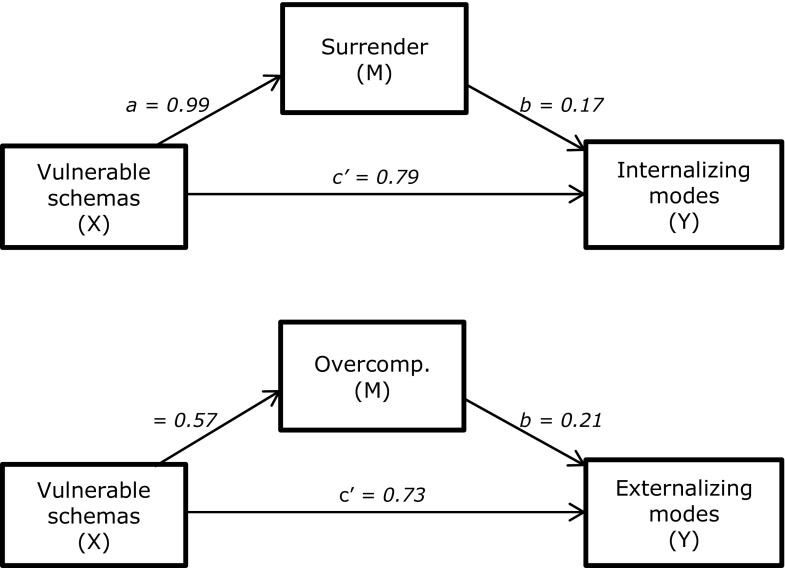
Simple mediation models estimating the direct and indirect (through coping) effects of Disconnection and Rejection EMS on Internalizing modes and on Externalizing modes. All path coefficients were significant at the 0.001 significance level

For all hypothesized relations between EMS and schema modes, we found significant indirect effects through coping responses. Thus, consistent with the findings in an adult population, schema coping mediated the relationship between EMS and schema modes in our adolescent sample. Effect sizes were largest in analyses with Surrender as a mediating variable, and smallest in analyses with Overcompensation as a mediating variable. In all mediation models, the effects of EMS on coping (path *a*) were statistically significant (ranging from 0.54 to 0.78, *M* = 0.66), as well as the effects of coping on schema modes (path *b*; ranging from 0.16 to 0.46, *M* = 0.26). Besides the indirect effects of EMS on schema modes through coping, we found evidence for direct effects, suggesting that EMS influenced schema modes also independent of schema coping. We also found significant indirect and direct effects for the models with higher-order variables. Path coefficients of these mediation models are depicted in Fig. 2.

#### Step 2: Simple Moderation Analyses

We found a moderation role for Surrender and Avoidance, but not Overcompensation, in some relationships between EMS and specific schema modes. More specifically, higher levels of surrendering coping led to a larger effect of all EMS on the Abandoned Child mode, and higher levels of avoidant coping led to a larger effect of all EMS on the Detached Protector mode. Although these interaction effects were statistically significant, the increase in *R*^*2*^ was very small (varying from 0.01 to 0.03). Note that in the model with higher-order EMS and schema mode variables, moderation failed to reach significance at the 0.001 level.

#### Step 3: Moderated Mediation

For those relationships between EMS and schema modes for which evidence of both a mediation and moderation role of coping was found, we tested moderated mediation models. In all but one hypothesized relationship, we found evidence for this more complex role of coping responses, as the index of moderated mediation was significantly different from zero (i.e., the corresponding 99.9% bias-corrected bootstrap confidence intervals did not straddle zero). Note that we did not investigate moderated mediation models for the higher-order variables of EMS and schema modes, because moderation analyses with these variables failed to reach significance.

### Role of Coping and Schema Modes in the Effect of EMS on Behavior Problems

#### Step 4: Multiple Mediation Analyses

In Step 4, we added internalizing and externalizing behavior problems to the models involving higher-order EMS and schema mode variables. Because we found evidence only for a mediating, not a moderating role of schema coping in the higher-order analyses, we treated the coping variables in Step 4 as mediators and not moderators. Thus, the effect of Disconnection and Rejection EMS on behavior problems was hypothesized to be mediated by coping and/or schema modes.

#### Internalizing Behavior Problems

**Fig. 3:**
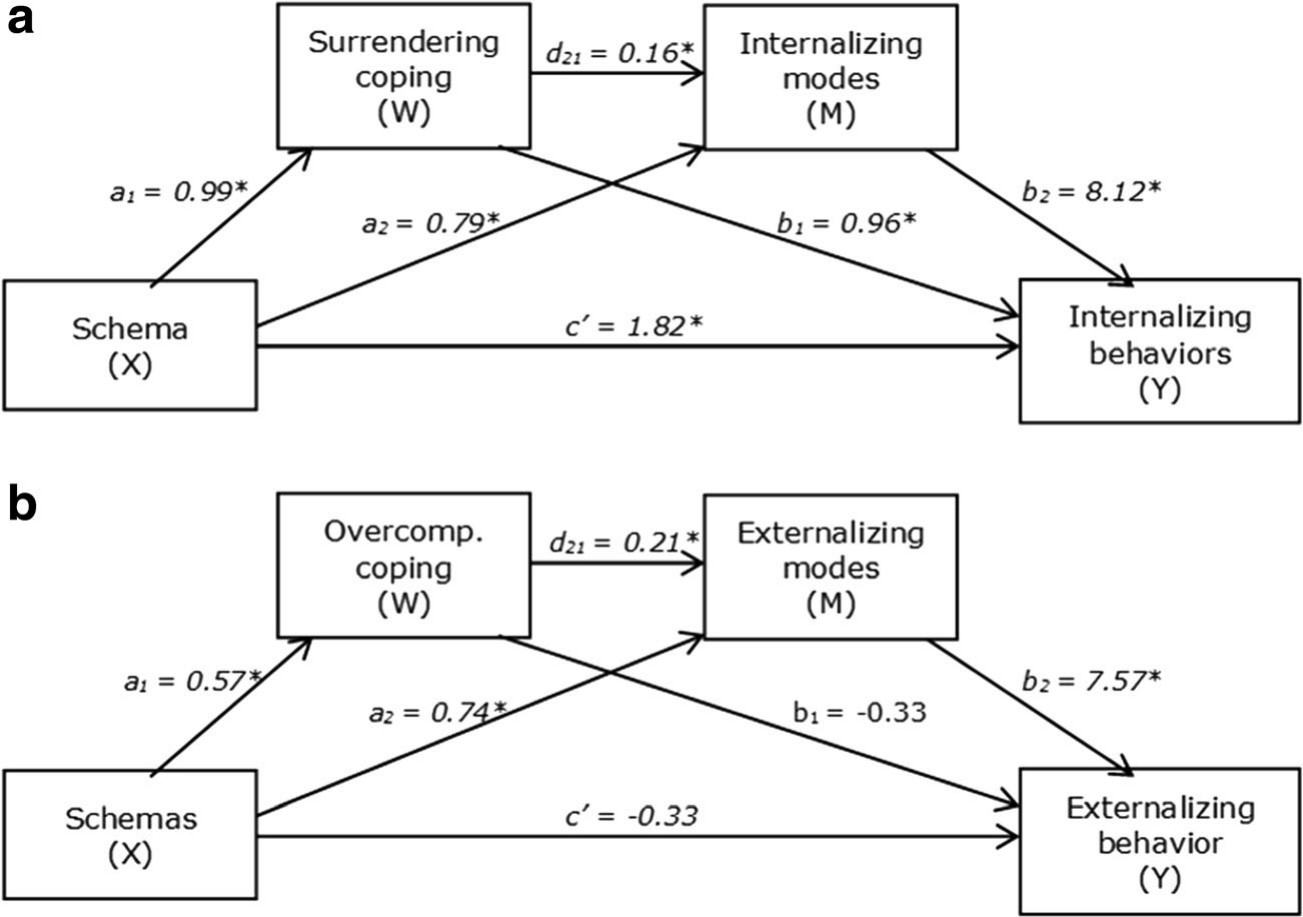
Multiple serial mediation models estimating the direct and indirect effects of Disconnection and Rejection EMS on Internalizing behavior problems (3**a**) and on Externalizing behavior problems (3**b**) through coping and schema modes. Statistically significant (<0.001) path coefficients are indicated with an asterisk (*)

From a serial multiple mediation analysis, EMS indirectly influenced Internalizing behavior problems through its effects on surrendering coping and Internalizing modes. As can be seen in Table 4 and Fig. 3a, EMS influenced surrendering coping (*a*_*1*_), surrendering coping influenced Internalizing modes (*d*_*21*_), and Internalizing modes influenced Internalizing behavior problems (*b*_*2*_). A 99.9% bias-corrected bootstrap confidence interval (BCI) based on 50,000 bootstrap samples for this indirect effect was entirely above zero, *a*_*1*_*d*_*21*_*b*_*2*_ = 1.34, 99.9% BCI [0.71, 2.13]. The completely standardized indirect effect (CSE) for *a*_*1*_*d*_*21*_*b*_*2*_ was 0.09. The results also showed that the two mediators, after controlling for each other, independently mediated the effect of EMS on Internalizing behavior problems. The bias-corrected bootstrap confidence interval for the indirect effect of EMS on Internalizing behavior problems through surrendering coping, independent on Internalizing modes, was entirely above zero, *a*_*1*_*b*_*1*_ = 0.95, CSE = 0.07, 99.9% BCI [0.07, 1.90]. The same was true for the confidence interval for the indirect effect *a*_*2*_*b*_*2*_ through Internalizing modes, independent of surrendering coping, *a*_*2*_*b*_*2*_ = 6.43, CSE = 0.45, 99.9% BCI [4.74, 8.44]. There were differences between the indirect effects *a*_*1*_*b*_*1*_*, a2b2,* and *a1d21b2*: The indirect effect through surrendering coping (*a*_*1*_*b*_*1*_) was smaller than the indirect effect through both surrendering coping and Internalizing modes (*a*_*1*_*d*_*21*_*b*_*2*_), 99.9% BCI [−7.88, −3.27]), which in turn was smaller than the indirect effect through Internalizing modes (*a*_*2*_*b*_*2*_), 99.9% BCI [−7.36, −3.21]. Besides the indirect effects, we found evidence that EMS had a direct effect on Internalizing behavior problems independent of surrendering coping and Internalizing modes, *c’* = 1.82, 99.9% BCI [0.05, 3.58]).

**Table 4:**
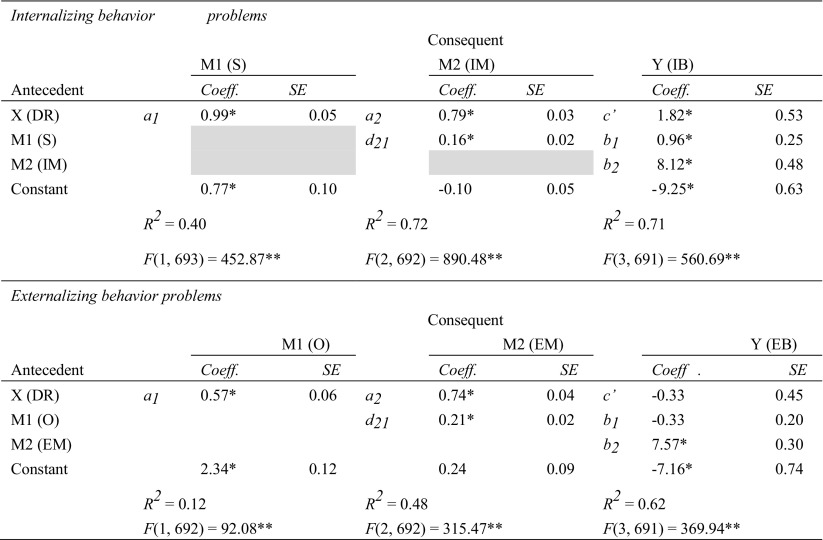
Results from the multiple mediation models investigating the direct and indirect effects of EMS on internalizing versus externalizing behavior problems

DR = Disconnection and rejection EMS; S = surrendering coping; IM = internalizing modes; EM = Externalizing modes; IB = internalizing behavior problems; EB = externalizing behavior problems

*p < 0.001

#### Externalizing Behavior Problems

For externalizing behavior problems, the results of the serial multiple mediation analysis are displayed in Table 4 and Fig. 3b. Disconnection and rejection EMS indirectly influenced Externalizing behavior problems through both overcompensatory coping and Externalizing modes. As shown in Fig. 3b, EMS showed an effect on Overcompensatory coping (*a*_*1*_), Overcompensatory coping affected Externalizing modes (*d*_*21*_), and Externalizing modes affected Externalizing behavior problems (*b*_*2*_). This indirect effect was significantly different from zero, *a*_*1*_*d*_*21*_*b*_*2*_ = 0.92, CSE = 0.08, 99.9% BCI [0.49, 1.57]. After controlling for Externalizing modes, we found no evidence for an indirect effect of EMS on Externalizing behavior problems through overcompensatory coping, *a*_*1*_*b*_*1*_ = −0.19, 99.9% BCI [−0.66, 0.21]. On the contrary, we found a significant indirect effect through Externalizing modes after controlling for overcompensatory coping, *a*_*2*_*b*_*2*_ = 5.57, CSE = 0.45, BCI [4.15, 7.28]. The indirect effect through both overcompensatory coping and Externalizing modes (*a*_*1*_*d*_*21*_*b*_*2*_) was smaller than the one through externalizing modes alone (*a*_*2*_*b*_*2*_), 99.9% BCI [−6.41, −3.08]. There was no evidence of a direct effect of schemas on Externalizing behavior problems independent on overcompensatory coping and externalizing modes, c’ = −0.33, 99.9% BCI [−1.58, 0.92].

## Discussion

This study investigated various models to explore the relationships between EMS regarding experiences of disconnection and rejection, coping responses, schema modes, and internalizing and externalizing behavior problems. We found clear evidence for a mediating role of schema coping in the relationships between EMS and schema modes, whereas the evidence for a moderating role of schema coping was much less convincing. The relationships between EMS, schema coping, and schema modes were consistent across the non-referred and clinical samples. Furthermore, this study demonstrated that EMS regarding experiences of disconnection and rejection predicted both internalizing and externalizing behavior problems in adolescents, and that schema coping and schema modes mediated these relationships.

### Mediation

With our single mediation analyses of specific EMS, coping, and schema modes, we replicated the findings of Rijkeboer and Lobbestael (2012). This evidence for mediation suggests that coping responses are the mechanisms through which EMS influence schema modes. Rijkeboer and Lobbestael (2012) have kindly provided us with the unstandardized regression coefficients and proportions of explained variance for each specific combination in their adult sample, so that we were able to directly compare these to the unstandardized regression coefficients in our adolescent sample. Overall, it seems that the mediation analyses involving overcompensatory coping as a mediator resulted in comparable proportions of explained variance and indirect effects in the adult (*ab* = 0.06–0.10) and adolescent samples (*ab* = 0.06–0.16). The mediation analyses involving avoidant coping seem to have resulted in comparable proportions of explained variance, but the indirect effects seem somewhat larger in the adolescent sample (*ab =* 0.12–0.18) compared to the adult sample (*ab* = 0.06–0.15). Finally, the mediation analyses involving surrendering coping seem to have produced larger proportions of explained variance in the adult sample than in the adolescent sample, whereas the indirect effects seem larger in the adolescent sample (*ab* = 0.18–0.22) compared to the adult sample (*ab* = 0.05–0.18). Note that these comparisons are observational; We did not statistically test for differences between the adult and adolescent samples. Thus, we do not know whether there are true differences in explained variance and effect sizes between these adolescent and adult samples, let alone if these differences are generalizable to the adolescent and adult population. More research is needed to clarify these issues, and to explore the implications of possible differences.

### Moderation and Moderated Mediation

We found only weak (increases in R^2^ ≤ 0.03) and inconsistent evidence that relationships between EMS and schema modes are dependent on the degree of various coping responses. In all but one of the models that showed evidence for moderation, we also found evidence for a more complex model of moderated mediation. This suggests that although the mediating mechanism of schema coping is most evident, some hypothesized relationships between EMS and schema modes are also dependent on the level of schema coping styles.

### Models for Schema Theory Constructs and Internalizing versus Externalizing Problems

Multiple mediation models showed that EMS predict both internalizing and externalizing behavior problems through mechanisms of schema coping and schema modes. This suggests that when such EMS are activated in adolescents, different coping responses and schema modes lead to different behavioral outcomes. As predicted, in the pathways to internalizing behavior problems, the EMS were associated with surrendering coping, leading to internalizing modes, which in turn were associated with internalizing behavior problems. In the pathways to externalizing behavior problems, the EMS were associated with overcompensatory coping, leading to externalizing modes, which in turn were associated with externalizing behavior problems.

Indirect effects of EMS on behavior problems were stronger via schema modes than via coping responses. This suggests that schema modes are more important than coping responses in explaining the effects of EMS on behavior problems. However, the differences in indirect effects via coping responses and via schema modes may also be due to the nature of these constructs. Schema modes consist of emotions, cognitions, and behaviors, and therefore partially overlap with the construct of coping responses. Hence, after controlling for the aspects of coping responses in schema modes, little unique variance may remain for modelling the indirect effect via coping responses.

The prominent mediating role of schema modes in the relationship between EMS and behavior problems underlines the importance of the schema mode construct in schema theory. Originally, schema theory included only EMS and coping. Schema modes were introduced because some patients with complex personality disorders (e.g., Borderline Personality Disorder) displayed extensive combinations of EMS and coping responses (Young et al. 2003). This study confirms that fixed combinations of EMS and coping responses result in specific schema modes, and that schema modes are important in explaining how EMS lead to adolescents’ behavior problems.

### Clinical Implications

The finding that the same EMS statistically predict both internalizing and externalizing behaviors supports the idea that externalizing behaviors are just as much a manifestation of EMS arising from experiences of disconnection and rejection, as are internalizing behaviors. Hence, a focus on EMS, coping, and schema modes (as in Schema Therapy; Young et al. 2003) may be a good choice of treatment for internalizing and externalizing behavior problems. Nonetheless, whether or not the current Schema Therapy techniques are effective in an adolescent population is another question, which deserves careful empirical tests. Several studies have found Schema Therapy to be effective for patients with personality disorders (Farrell et al. 2009; Giesen-Bloo et al. 2006; Nadort et al. 2009), both in samples including patients with internalizing behaviors (Bamelis et al. 2014) and externalizing behaviors (Bernstein et al., Effectiveness of Schema therapy versus treatment-as-usual for forensic inpatients with personality disorders: A randomized clinical trial, unpublished manuscript). Effect sizes were medium to large with respect to changes in EMS/schema modes and symptoms. Recent studies have provided preliminary evidence for the effectiveness of Schema Therapy in adolescent patients with personality disorder traits and internalizing, mood problems (Roelofs et al. 2016) and externalizing, disruptive behaviors (Van Wijk-Herbrink et al. 2017a). We are currently conducting a randomized controlled trial on adolescents in residential treatment for externalizing behavior problems. This and other studies will shed light on whether Schema Therapy is indeed an effective treatment for internalizing and externalizing behavior problems in adolescents.

### Strengths, Limitations, and Future Research

Strengths of the present study are the relatively large sample size and the use of a mixed non-referred and clinical sample. Of course, this study also has some limitations, such as its cross-sectional design. Therefore, all relationships in the models were susceptible to confounding and epiphenomenal associations. Furthermore, although schema theory clearly guided the causal order of the schema-related constructs that we modelled, methodologically we cannot rule out other order effects. We cannot make inferences about the causality of the relationships. Subsequently, we cannot conclude that intervening to change EMS, coping, and schema modes, for example with Schema Therapy, will change adolescents’ behavior problems. Treatment studies investigating mechanisms of change (see Kazdin and Nock 2003) should focus on this. Another limitation is that we relied solely on self-report questionnaires, which are limited by methodological factors such as response biases. Nevertheless, self-report measures also have an important role to play, because they tell us about the subjective experience of schema theory constructs and behavior problems. Most evidence for the psychometric properties of the questionnaires are from adult samples, although quite good evidence also exist for the use of the YSQ and SMI in adolescents. We know less about the psychometric properties of the SCI due to its’ fairly recent development, which is a limitation of this study. If the reliability and validity of this measure would be weak, it could potentially attenuate relationships. So, if anything, this would make it harder to detect significant relationships, whereas in our study most hypotheses were confirmed.

Although the response rate in the clinical sample was rather good considering the oppositional tendencies of this population, there was a relatively low response rate in the non-referred sample. We do not know the reasons for not participating in this study (non-referred sample) or not completing the questionnaires (clinical sample), and have no way of comparing the participants to the non-participants, which is a limitation of this study. It may be that non-responders from the clinical sample refused to fill out the questionnaires (although it could also be that staff simply forgot to administer them), and that this relates to their severity of oppositional behaviors. We can only speculate, but if this were true, then we cannot know for sure whether the mechanisms found in this study will also hold up for patients with most severe oppositional behaviors. Although severity of behavior problems did not seem to affect the mechanisms (according to the non-significant differences between the clinical and non-referred sample) and theoretically, we have no reason to assume that this would be any different for extreme oppositional adolescents, we cannot entirely rule out this possibility. Finally, as we have a research program investigating schema theory constructs, we are of course subject to possible biases in favor of our own hypotheses. Therefore, we took precautions to try and mitigate any self-serving biases, for example by having very specific hypotheses and by setting a stringent significance level for model testing.

Nonetheless, our findings need to be replicated in other (independent) studies. Future research should aim to replicate our results in longitudinal research designs, using a combination of self-report, other-report, and observational measures. Also, it would be interesting to investigate whether the constructs of EMS and schema modes have measurement invariance in clinical and non-referred samples, as was demonstrated for schema coping (Van Wijk-Herbrink et al. 2017b).

### Conclusion

In conclusion, this study confirms theorized associations between schema theory constructs of EMS, coping responses, and schema modes, and clarifies important aspects of the nature of these relationships. It suggests that adolescents both with internalizing behaviors and with externalizing behaviors could possibly benefit from Schema Therapy targeting EMS related to experiences of disconnection and rejection. The effectiveness of Schema Therapy with adolescents, therefore, deserves further study.

## Footnotes

1 The exact amount of analyses was dependent on whether or not moderated mediation analyses would logically follow from the results of the simple mediation and simple moderation analyses

2 One exception was found with respect to the interaction effect of Abandonment x overcompensation on the Angry Child mode. This significant group difference was rather meaningless, though, because the interaction effect was not significant in either group.
